# Clinical, molecular, metabolic, and immune features associated with oxidative phosphorylation in melanoma brain metastases

**DOI:** 10.1093/noajnl/vdaa177

**Published:** 2021-01-06

**Authors:** Grant M Fischer, Renato A Guerrieri, Qianghua Hu, Aron Y Joon, Swaminathan Kumar, Lauren E Haydu, Jennifer L McQuade, Y N Vashisht Gopal, Barbara Knighton, Wanleng Deng, Courtney W Hudgens, Alexander J Lazar, Michael T Tetzlaff, Michael A Davies

**Affiliations:** 1 Department of Translational Molecular Pathology, The University of Texas MD Anderson Cancer Center, Houston, Texas, USA; 2 Department of Melanoma Medical Oncology, The University of Texas MD Anderson Cancer Center, Houston, Texas, USA; 3 Department of Biostatistics, The University of Texas MD Anderson Cancer Center, Houston, Texas, USA; 4 Department of Surgical Oncology, The University of Texas MD Anderson Cancer Center, Houston, Texas, USA; 5 Department of Pathology/Lab Medicine, The University of Texas MD Anderson Cancer Center, Houston, Texas, USA; 6 Department of Genomic Medicine, The University of Texas MD Anderson Cancer Center, Houston, Texas, USA; 7 Department of Systems Biology, The University of Texas MD Anderson Cancer Center, Houston, Texas, USA

**Keywords:** brain metastases, immune therapy, melanoma, oxidative phosphorylation, targeted therapy

## Abstract

**Background:**

Recently, we showed that melanoma brain metastases (MBMs) are characterized by increased utilization of the oxidative phosphorylation (OXPHOS) metabolic pathway compared to melanoma extracranial metastases (ECMs). MBM growth was inhibited by a potent direct OXPHOS inhibitor, but observed toxicities support the need to identify alternative therapeutic strategies. Thus, we explored the features associated with OXPHOS to improve our understanding of the pathogenesis and potential therapeutic vulnerabilities of MBMs.

**Methods:**

We applied an OXPHOS gene signature to our cohort of surgically resected MBMs that had undergone RNA-sequencing (RNA-seq) (*n* = 88). Clustering by curated gene sets identified MBMs with significant enrichment (High-OXPHOS; *n* = 21) and depletion (Low-OXPHOS; *n* = 25) of OXPHOS genes. Clinical data, RNA-seq analysis, and immunohistochemistry were utilized to identify significant clinical, molecular, metabolic, and immune associations with OXPHOS in MBMs. Preclinical models were used to further compare melanomas with High- and Low-OXPHOS and for functional validation.

**Results:**

High-OXPHOS MBMs were associated with shorter survival from craniotomy compared to Low-OXPHOS MBMs. High-OXPHOS MBMs exhibited an increase in glutamine metabolism, and treatment with the glutaminase inhibitor CB839 improved survival in mice with MAPKi-resistant, High-OXPHOS intracranial xenografts. High-OXPHOS MBMs also exhibited a transcriptional signature of deficient immune activation, which was reversed in B16-F10 intracranial tumors with metformin treatment, an OXPHOS inhibitor.

**Conclusions:**

OXPHOS is associated with distinct clinical, molecular, metabolic, and immune phenotypes in MBMs. These associations suggest rational therapeutic strategies for further testing to improve outcomes in MBM patients.

Key Points

- OXPHOS correlates with shorter survival in melanoma brain metastasis (MBM) patients.
- OXPHOS associates with distinct molecular, metabolic, and immune features in MBMs.
- Targeting OXPHOS-associated features could improve treatment outcomes for MBMs.

**Importance of the Study**

We have recently demonstrated that increased oxidative phosphorylation (OXPHOS) is a hallmark and functional dependency of melanoma brain metastases (MBMs). However, little is known about the contribution of OXPHOS to the pathogenesis of MBMs, which could inform the rational development of new therapeutic strategies for affected patients. As OXPHOS levels are heterogeneous among MBMs, we compared High- and Low-OXPHOS tumors to identify features significantly associated with this metabolic phenotype. Our results show that High-OXPHOS in MBMs is associated with shorter survival, increased mTOR activation, increased utilization of glutamine metabolism, and an immunosuppressed tumor microenvironment. Our studies in preclinical models support the functional nature and importance of these metabolic and immune associations. Together the results provide new information about the pathogenesis of OXPHOS in MBMs, and they suggest potential new therapeutic strategies for these aggressive tumors.

Melanoma is the most aggressive of the common forms of skin cancer, and has among the highest risk of brain metastasis among all solid tumors.^1,2^ Despite many advances in systemic treatments, brain metastases remain a critical challenge to overcome to further improve patient outcomes. For example, the COMBI-MB trial showed that although the FDA-approved targeted therapy regimen of dabrafenib and trametinib achieves intracranial responses in ~60% of patients with melanoma brain metastases (MBMs), the duration of response was ~50% shorter than that achieved in melanoma extracranial metastases (ECMs).^3^ Further, the FDA-approved anti-PD1 antibodies pembrolizumab and nivolumab achieve clinical responses in only ~20% of patients with MBMs, which is much lower than the response rate (~40%) in patients without CNS involvement.^4,5^ A more promising intracranial benefit rate (58.4%) was achieved with ipilimumab and nivolumab combination immunotherapy in patients with asymptomatic MBMs, but the intracranial benefit rate was only 22.2% in patients with symptomatic tumors.^6,7^ Thus, there remains an unmet need to develop new approaches to more effectively prevent and/or treat MBMs.

We recently reported the first RNA-sequencing (RNA-seq) analysis of melanoma patients that underwent surgical removal of both MBMs and ECMs. This RNA-seq analysis showed that MBMs were characterized by increased expression of genes involved in the oxidative phosphorylation (OXPHOS) metabolic pathway, and decreased immune cell infiltrates, compared to patient-matched ECMs.^8^ We and others have previously shown that OXPHOS mediates de novo and acquired resistance to FDA-approved MAPK pathway inhibitors.^9–11^ More recently, OXPHOS has also been implicated in resistance to anti-PD1 immunotherapy.^12,13^ Importantly, we showed that treatment with IACS-010759, a potent direct OXPHOS inhibitor, significantly improved the survival of mice with intracranial melanoma xenografts, supporting the functional significance of OXPHOS in MBMs.^8^ However, IACS-010759 has been associated with significant toxicity in preclinical models and in early-phase clinical trials.^14,15^ Thus, there is a need to identify alternative strategies to overcome this metabolic pathway’s negative effects.

Here, we have used our unique cohort of characterized MBMs, and melanoma preclinical models, to identify clinical, molecular, immune, and metabolic features that are associated with OXPHOS. These studies provide new insights into the pathogenesis of OXPHOS in MBMs. Our results also suggest potential new therapeutic strategies for this common and deadly manifestation of metastatic melanoma.

## Materials and Methods

### Cell Lines

All cell lines were grown at 37°C under 5% CO_2_. Luciferase-tagged A375, MEL624, and A375-R1 cells were grown in RPMI-1640 media supplemented with glutamine and heat-inactivated 5% fetal bovine serum (FBS) (Gibco). Luciferase-tagged B16-F10 cells (developed and provided by Dr. Willem Overwijk, MD Anderson) were grown in RPMI-1640 media supplemented with glutamine and 10% heat-inactivated FBS. Identity of the human cell lines was verified by short-tandem repeat (STR) fingerprinting at least every 6 months.^16^ All cell lines were confirmed negative for mycoplasma prior to the study using the MycoAlert Mycoplasma Detection Kit (Lonza) according to the manufacturer’s specifications. Species, mutations, and OXPHOS status of the cell lines are presented in Supplementary Table S1.

### Compounds

The following compounds were acquired: metformin (Cayman Chemical), isotype rat IgG control antibody (BioXCell, Clone: 2A3, #BE0089), anti-mouse PD1 antibody (BioXCell, Clone: RMP1-14, #BE0146), and AZD2014 (Selleck Chemical), and CB839 (Selleck Chemical). Compounds were formulated as previously described.^13,15,17^

### Mice

All mouse experiments were approved by the Institutional Animal Care and Use Committee. Female C57BL/6 and CD-1 nude mice were purchased from the Jackson Laboratory and Charles River Laboratory, respectively. Experiments using C57BL/6 and CD-1 nude mice at 8 weeks of age, housed in specific pathogen-free conditions, were performed at the MD Anderson South Campus Animal Vivarium.

### Stereotactic Intracranial Injection

Intracranial (ICr) tumors were established by direct injection, and monitored by bioluminescence imaging (BLI), in C57BL/6 mice (B16-F10) or CD-1 nude mice (A375, A375-R1, and MEL624) as previously described.^18^ Harvested tumors were washed briefly in ice-cold normal saline and either flash frozen in liquid nitrogen or fixed in formalin overnight, then dehydrated in 70% ethyl alcohol, and paraffin embedded.

### RNA sequencing and Whole-Exome Sequencing Analyses

Raw counts and WES BAM files were acquired, and the OXPHOS-Index (OP-Index) was derived, as previously described.^8^ Comparisons of interest were performed using functions from the *edgeR* and *limma/voom* Bioconductor packages in R (v3.6.1).^19^ ssGSEA was conducted on TMM-normalized, voom-transformed log2-(CPM+0.5) expression matrices using the GenePattern module *ssGSEAProjection* (v9.0.10) with default settings to generate enrichment scores for the 8 OXPHOS-related gene sets listed in Supplementary Table S2. Values from the 8 components of the OP-Indices of MBMs were median centered. Hierarchical clustering was conducted with distances calculated using Euclidean correlation metrics and clusters joined using complete linkage. Preranked GSEA (GSEA-P) was performed as previously described.^8^ CIBERSORT was run in “absolute mode” to estimate intratumoral immune cell populations from linear RPKM values, as previously described.^20^ Values were log2-transformed and median-centered for heatmap generation. All heatmaps were generated via ClustVis (https://biit.cs.ut.ee/clustvis/). MuTect 1.1.4 was used to detect potential single-nucleotide variations for samples with available WES data. In addition to the build-in filter, we included variants with allele frequency in tumor DNA ≥2% and allele frequency in germline DNA ≤ 2%, as well as total read number in tumor > 20 and total read number in normal tissue >10.

### Targeted Metabolomics

Mice bearing ICr A375 and A375-R1 tumors were euthanized once moribund. Harvested tumors were frozen in liquid nitrogen and submitted to the Baylor College of Medicine Metabolomics Core. Sample preparation and data acquisition for 46 metabolites (glycolytic/TCA cycle intermediates, amino acids, fatty acids, NADH, and ATP) are previously described.^8^ Normalized data were log2-transformed. For every metabolite in the normalized dataset, paired Student’s *t*-tests were conducted to compare expression levels between groups. Differential metabolites were identified by adjusting the *P*-values for multiple testing at a false discovery rate (FDR) threshold of <0.25, as previously described.^21^ All metabolites significantly upregulated in A375-R1 ICr xenografts (log2FC > 0 and FDR q-val < 0.25) were uploaded into MetaboAnalyst 4.0 (http://www.metaboanalyst.ca/). The Pathway Analysis tool set to default settings was used to perform overrepresentation analysis of the significantly upregulated metabolites, as previously described.^22^

### Therapeutic Studies

Mice were weighed every 2 days and were euthanized if 20% weight loss occurred, or if mice were moribund or displaying neurological symptoms (ataxia, seizures, circling behavior, paralysis, or cranial doming). Treatments were designed to end 42 days after randomization unless otherwise specified.

#### AZD2014 efficacy analysis

1 × 10^3^ luciferase-tagged A375-R1 cells suspended in Hank’s Buffered Saline Solution (HBSS; Corning, Inc.) were directly implanted in the brain parenchyma of CD-1 nude mice. After 7 days, tumor uptake was confirmed via BLI and mice were randomized into 2 treatment arms: vehicle or AZD2014 (20 mg/kg p.o. once daily).

#### AZD2014 pharmacodynamics (PD) analysis

CD-1 nude mice bearing ICr A375-R1 xenograft tumors were treated with vehicle or AZD2014 (20 mg/kg p.o. once daily). On day 7, mice were given one final treatment. Three hours after this treatment, tumors were harvested and fixed in 10% formalin. FFPE slides of these tumors were generated and probed for P-S6 (marker of mTOR pathway activation).

#### Metformin+anti-PD1

5 × 10^3^ luciferase-tagged B16-F10 cells suspended in HBSS were directly implanted in the brain parenchyma of C57BL/6 mice. After 5 days, tumor uptake was confirmed via BLI and mice were randomized into 4 treatment arms: isotype antibody (ab) control (200 µg i.p. 3×/week) + PBS (10 µL/g body weight i.p. every other day); anti-PD1 ab (200 µg i.p. 3×/week) + PBS (10 µL/g body weight i.p. every other day); isotype ab control (200 µg i.p. 3×/week) + metformin (50 mg/kg i.p. every other day); and anti-PD1 ab (200 µg i.p. 3×/week) + metformin (50 mg/kg i.p. every other day).

#### CB839 monotherapy

Cell proliferation inhibition was determined using Cell Titer Blue (Promega), as previously described. 9.1 × 10^3^ luciferase-tagged A375-R1 and 1 × 10^4^ MEL624 cells suspended in HBSS were directly implanted in the brain parenchyma of CD-1 nude mice. After 7 days, tumor uptake was confirmed via BLI, and mice were randomized to vehicle and CB839 (200 mg/kg p.o. twice daily) treatment groups.

### qRT-PCR Analysis

#### RNA extraction and cDNA synthesis

The Roche High Pure miRNA kit was used according to the manufacturer’s specifications for RNA extraction from B16-F10 ICr xenografts extracted and frozen after 96 h of treatment with PBS (10 uL/g body weight i.p. every other day) or metformin (50 mg/kg i.p. every other day). One thousand nanograms of RNA was used to synthesize the first strand of cDNA using the High Capacity cDNA Archive kit (Applied Biosystems) following standard ABI Protocol.

#### qRT-PCR

Inventoried TaqMan assays were purchased from Life Technologies [Mm00443258_m1 (*Tnf*), Mm00812512_m1 (*Prf1*), Mm01168134_m1 (*Ifng*), Mm00442837_m1 (*Gzmb*), Mm01182107_g1 (*Cd8a*), Mm00445235_m1 (*Cxcl10*), Mm00434946_m1 (*Cxcl9*), Mm00492586_m1 (*Ido1*), Mm00439531_m1 (*Stat1*)]. All qRT-PCR reactions were performed using the 7900HT Fast Real-Time PCR system and Taqman gene expression master mix (Applied Biosystems) with a standard cycling program of 40 cycles at 95°C for 15 s and at 60°C for 1 min. All reactions were run in triplicate and normalized to human *18S* (Hs99999901_s1). Data were analyzed using the 2^-ΔΔ*CT*^ method.

### Bioenergetics Stress Test

A Seahorse XFe96 Bioanalyzer (Agilent) was used to acquire oxygen consumption rate (OCR) and extracellular acidification rate (ECAR) values as previously described.^9^ Data were normalized against cell numbers.

### Immunohistochemistry

All IHC studies were performed on 5 µm FFPE sections using a Leica BOND RXm autostainer. Slides were stained with antibodies targeting human P-S6 (Cell Signaling #4858, 1:100) and P-PRAS40 (Cell Signaling #13175, 1:200) using a modified version of either the standard Leica Bond DAB “F” or red “J” IHC protocols. Slides were scored by a board-certified pathologist and given an H-score based on percentage and intensity of positivity. CD3, CD8, PAX5, and PTEN staining and analyses were performed as previously described.^8^

### Statistical Analyses

Overall survival (OS) was defined as the time interval from date of craniotomy (clinical samples) or treatment initiation (mouse studies) to censoring or date of death from any cause. Survival duration was analyzed by the Kaplan–Meier method. Survival curves were drawn in Prism 8.0 (GraphPad Software). Hazard ratios and significance were calculated via the Mantel-Haenszel test and log-rank test, respectively, in Prism 8.0 (GraphPad Software). Additional data analyses and representations were performed either with R (v3.6.1), Microsoft Excel 2013, or Prism 8.0 (GraphPad Software). Comparison of continuous variables between 2 groups was performed by unpaired or paired Student’s *t*-test. The Pearson correlation coefficient was calculated to assess correlation between continuous variables. To control for multiple hypothesis testing, we applied the Benjamini-Hochberg method. Lastly, all statistical significance testing was 2-sided with a Type-I error rate of 0.05 except where specifically noted in relevant Figure legends.

## Results

### Heterogeneity and Clinical Association of OXPHOS in MBMs

**Figure 1.**
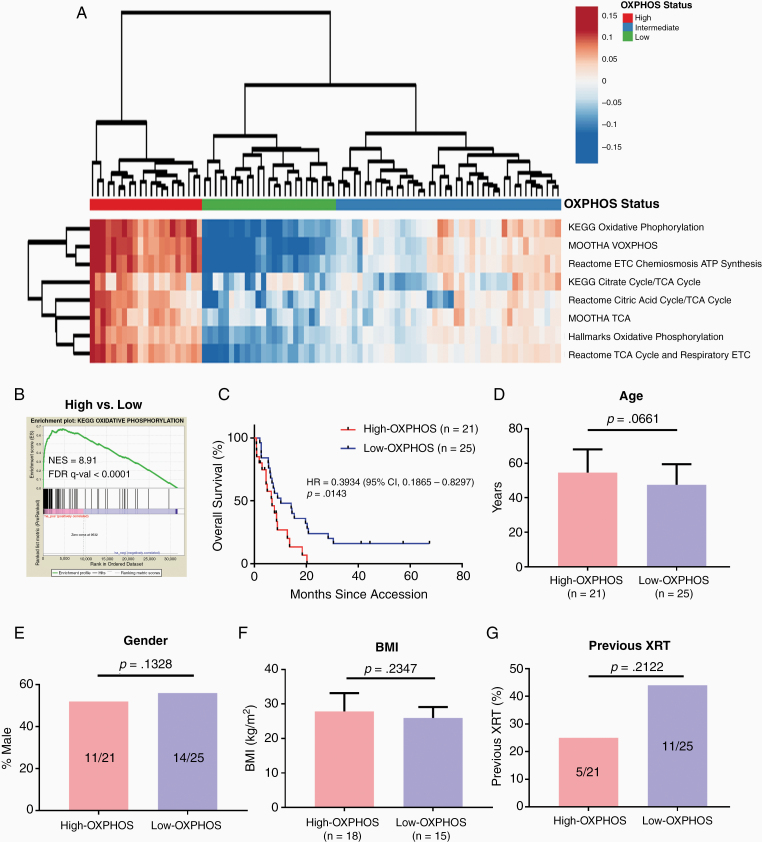
Identification of High-OXPHOS and Low-OXPHOS melanoma brain metastases. (A) Hierarchical clustering of the OXPHOS-Indices from 88 melanoma brain metastases (MBMs) with available RNA-seq data resulted in the formation of 3 clusters: MBMs with significant enrichment (High-OXPHOS; *n* = 21); depletion (Low-OXPHOS; *n* = 25); or intermediate (Intermediate-OXPHOS; *n* = 42) OXPHOS gene set enrichment. Results are represented as a heatmap of median-centered values from each of the 8 components of the OXPHOS-Index. (B) GSEA-P confirming enrichment of OXPHOS in MBMs identified as High-OXPHOS versus Low-OXPHOS. (C) Kaplan–Meier analysis of overall survival from craniotomy for patients with High-OXPHOS versus patients with Low-OXPHOS MBMs. Hazard ratio determined via Mantel-Haenszel test and significance by log-rank test. (D–G) Comparison of clinical variables between patients with High- and Low-OXPHOS MBMs, including mean age, gender, mean body mass index (BMI), and frequency of previous radiation XRT). Significance was determined for age and BMI via 2-sided Student’s *t*-test; gender and frequency of prior XRT were compared via 2-sided Fisher’s exact test.

In order to characterize the heterogeneity of OXPHOS among MBMs, we calculated the OXPHOS-Index (OP-Index) for each tumor in our previously described cohort of 88 MBMs with available RNA-seq data.^8^ Hierarchical clustering identified 3 clusters, which we termed “High-OXPHOS,” “Intermediate-OXPHOS,” and “Low-OXPHOS” (Figure 1A). Pre-ranked Gene Set Enrichment Analysis (GSEA-P) confirmed that the KEGG OXPHOS gene set was significantly enriched (FDR q-val < 0.0001) in High- versus Low-OXPHOS MBMs (Figure 1B).

We tested whether OXPHOS status was associated with clinical features of MBM patients. We compared the outcome of High-OXPHOS versus Low-OXPHOS MBMs for this and future analyses, as the difference in gene expression was most significant for these groups (Figure 1B and Supplementary Figure S1A–B). Supporting the clinical significance of the observed heterogeneity of OXPHOS among MBMs, patients with Low-OXPHOS MBMs had improved OS from craniotomy versus patients with High-OXPHOS (HR 0.393, 95% confidence interval [CI] 0.187–0.830, *P* = .0143; Figure 1C). In contrast to the association with clinical outcomes, review of clinical features showed that OXPHOS status was not associated significantly with age, gender, body mass index (BMI), or history of prior radiation therapy (XRT) (Figure 1D–G). There was also no significant association for OXPHOS status with history of prior systemic therapy (*P* = .23).

### Molecular Associations of High-OXPHOS in MBMs

**Figure 2.**
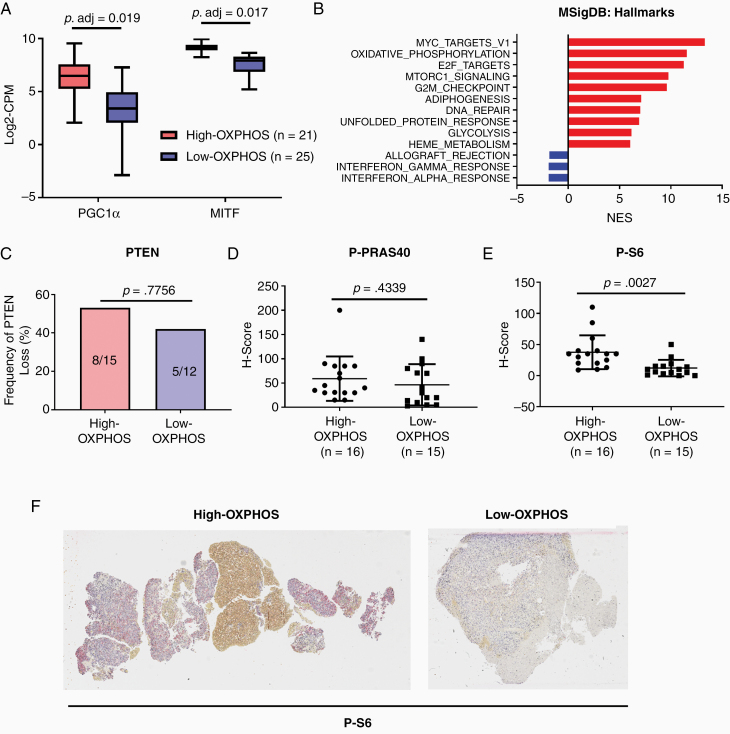
Molecular associations of OXPHOS in melanoma brain metastases. (A) Gene expression analysis of PGC1α and MITF (by RNAseq) in High-(Red) versus Low-OXPHOS (Blue) melanoma brain metastases (MBMs). Each plot is a simple box and whisker plot. Median values (lines) and interquartile range (whiskers) are indicated. Adjusted *P* values calculated via generalized linear model analysis are listed. (B) Cumulative GSEA-P enrichment plot demonstrating significant enrichment or depletion (false discovery rate [FDR] q-val < 0.0001) of MSigDB Hallmarks gene sets in High-OXPHOS versus Low-OXPHOS MBMS identified via clustering methods. The 10 most up-regulated gene sets are shown in red. All significantly down-regulated gene sets are shown in blue. The normalized enrichment score forms the x-axis. (C) Comparison of the prevalence of complete PTEN loss by IHC for High-OXPHOS and Low-OXPHOS MBMs. Y-axis represents the frequency (%) of MBMs with complete absence of PTEN expression. Significance determined via 2-sided Fisher’s exact test. (D) Comparison of P-PRAS40 IHC expression by H-scores for High-OXPHOS versus Low-OXPHOS MBMs. Lines represent mean ± SD; each dot represents a single tumor. Significance determined via 2-sided Student’s *t*-test. (E) Comparison of P-S6 IHC expression by H-scores for High-OXPHOS versus Low-OXPHOS MBMs. (F) Representative P-S6 staining in High- and Low-OXPHOS MBMs. Samples selected reflect the median H-scores in the High- (median = 35) and Low- (median = 10) OXPHOS MBMs. Tumor cells are present throughout the entirety of both samples.

As multiple pathways have been implicated in melanoma progression and therapeutic resistance, we next analyzed MBMs for molecular associations with OXPHOS. As anticipated, High-OXPHOS MBMs expressed higher levels of PGC1α, a transcriptional co-factor that is a critical regulator of many OXPHOS-related genes in melanoma,^9,11,23^ compared to Low-OXPHOS MBMs (*P*.adj = .0188; Figure 2A). High-OXPHOS MBMs also had higher expression of MITF, a lineage-specific transcription factor that regulates PGC1α in melanoma (*P*.adj = .0169; Figure 2A).^9,11,23^ Previously, we observed in human melanoma cell lines that MITF activity, and thus PGC1α expression, are regulated in melanoma by mTOR.^9^ Consistent with that result, GSEA-P analysis of High- versus Low-OXPHOS MBMs demonstrated significant enrichment (FDR q-val < 0.0001) of genes regulated by the mTORC1 signaling pathway in High-OXPHOS tumors (Figure 2B). As previous protein-based studies of MBMs by our group and others identified increased activation of the PI3K-AKT pathway in MBMs,^24,25^ we further evaluated the molecular associations of OXPHOS status in MBMs by performing IHC for PTEN (complete loss promotes PI3K-AKT pathway activity), P-PRAS40 (marker of AKT activity), and P-S6 (marker of mTORC1 activity). While we detected no significant difference in PTEN loss (*P* = .7756) or P-PRAS40 expression (*P* = .4339; Figure 2C and D), P-S6 expression was significantly increased (*P* = .0027) in High- versus Low-OXPHOS MBMs, suggesting association with mTOR activity, but not PI3K/AKT (Figure 2E and F). We did not detect any significant difference in the prevalence of activating *BRAF* mutations, nor in the mutation rate of 74 therapeutically targetable genes, between tumors with High- versus Low-OXPHOS (Supplementary Table S3).^26^

Based on the findings implicating increased mTOR signaling in High-OXPHOS MBMs, we evaluated the clinical efficacy of AZD2014, a mTORC1/2 inhibitor previously shown to completely ablate mTOR signaling and synergize with MEKi in subcutaneous High-OXPHOS melanoma xenografts.^9^ Mice bearing intrancranial xenografts of the High-OXPHOS A375-R1 cell line were randomized to treatment with AZD2014 (20 mg/kg p.o. once daily) or vehicle control. AZD2014 failed to improve OS relative to vehicle controls (Supplementary Figure S2A). Because AZD2014 works most effectively when combined with MEKi,^9^ we investigated if the lack of efficacy for A375-R1 intracranial xenografts was due to insufficient target inhibition or because this drug is simply inadequate as a single-agent strategy. Interestingly, 7 days of treatment with AZD2014 did not suppress P-S6 staining compared to vehicle controls (*P* = .8623), indicating that the drug failed to inhibit its target (Supplementary Figure S2B–C) in the intracranial xenografts, thus making it a poor candidate for further MBM therapeutics studies.

### Increased Glutamine Metabolism in High-OXPHOS MBMs

**Figure 3.**
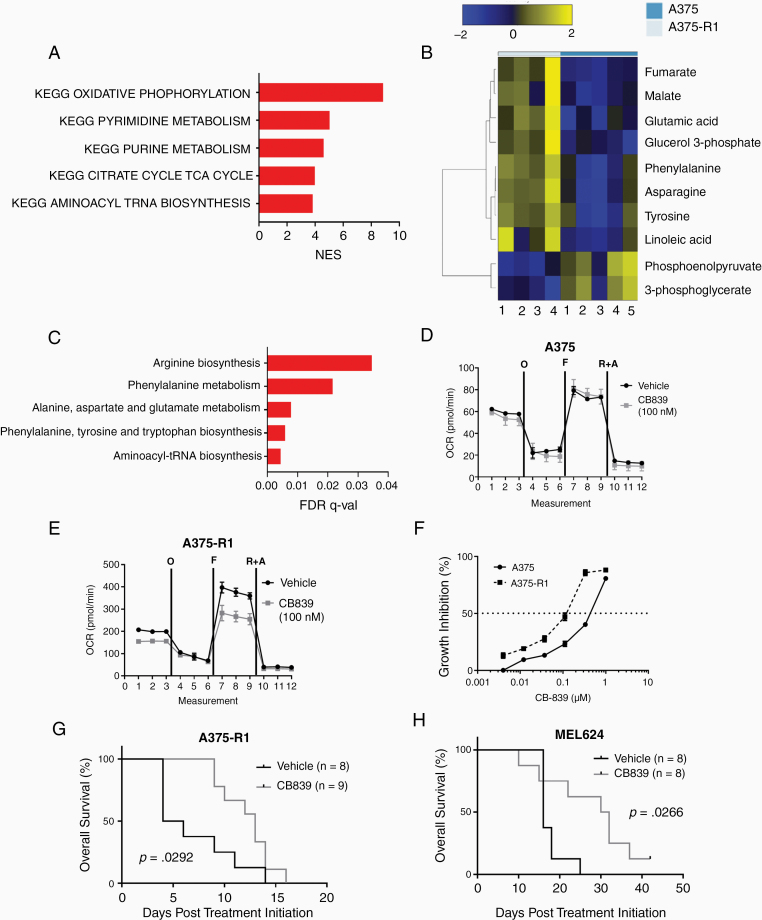
Metabolic profiling of High-OXPHOS melanoma brain metastases (MBMs) identifies glutamine metabolism as a therapeutic target. (A) Cumulative GSEA-P enrichment plot demonstrating significant enrichment or depletion (false discovery rate [FDR] q-val < 0.0001) of KEGG metabolism gene sets in High-OXPHOS versus Low-OXPHOS MBMS. Enriched gene sets are shown in red. No depleted gene sets met the criteria for significance. X-axis shows the normalized enrichment score for each pathway. (B) Differentially expressed metabolites (FDR q-val < 0.25) between A375 (Low-OXPHOS; *n* = 5) and A375-R1 (High-OXPHOS; *n* = 4) human melanoma intracranial xenografts, as determined by liquid chromatography mass spectrometry (LC-MS). Heatmap shows median-centered log2-tranformed concentrations of these metabolites. (C) Pathway analysis of metabolites significantly upregulated (log2FC>0 and FDR q-val < 0.25) in A375-R1 versus A375 intracranial xenografts. All pathways listed are significantly enriched in A375-R1 versus A375 (FDR q-val < 0.05). (D,E) Seahorse mitochondrial stress test results for A375 and A375-R1 cells treated for 12 h with vehicle or 100 nM of CB839 *in vitro*. The figures show basal, oligomycin-inhibited (“O”), FCCP-activated (“F”), and Antimycin/Rotenone-inhibited (“A&R”) oxygen consumption rate levels. Data are representative of quadruplicates and SD. (F) Cell proliferation inhibition of A375 and A375-R1 cell lines treated with CB839 for 72 h in vitro. Data are representative of triplicates and SD. (G) Kaplan–Meier analysis of overall survival (OS) for mice bearing intracranial A375-R1 xenografts treated with vehicle or CB839 (200 mg/kg p.o. twice daily). Significance was determined by log-rank testing. (H) Kaplan–Meier analysis of OS for mice bearing intracranial MEL624 xenografts treated with vehicle or CB839 (200 mg/kg p.o. twice daily). Significance determined by log-rank testing.

Oxidative phosphorylation interacts with numerous other metabolic pathways.^27^ Thus, to better understand the metabolic features of High-OXPHOS MBMs, we selected 70 MSigDB KEGG metabolism-specific gene sets (Supplementary Table S4) and performed GSEA-P on the High- versus Low-OXPHOS MBMs. As expected, the KEGG OXPHOS gene set was the most enriched pathway in the High-OXPHOS MBMs (FDR q-val < 0.0001; Figure 3A). Interestingly, the next most enriched pathways included purine synthesis, pyrimidine synthesis, and aminoacyl tRNA biosynthesis (Figure 3A). Enrichment of these nucleic acid synthesis and protein synthesis pathways suggests increased growth potential in High-OXPHOS MBMs. Importantly, all of these pathways require glutamine, which represents a potential therapeutic target.

Direct metabolite analysis was not feasible on the clinical samples, as they were all FFPE. Thus, we performed liquid chromatography-mass spectrometry (LC-MS) analysis on fresh A375 (*BRAF* mutant human melanoma, Low-OXPHOS) and A375-R1 (A375 subclone with acquired resistance to MAPKi and High-OXPHOS) intracranial xenografts to further explore the metabolic features of High- and Low-OXPHOS MBMs.^9^ As expected, A375-R1 MBMs had significantly higher concentrations of the TCA cycle metabolites malate and fumarate, and lower concentrations of the glycolytic metabolites phosphoenolpyruvate and 3-phosphoglycerate, versus A375 (Figure 3B). A375-R1 MBMs also had significantly increased concentrations of glutamic acid and asparagine, consistent with increased glutaminolysis (Figure 3B). Pathway analysis of differentially expressed metabolites identified significant enrichment (FDR q-val < 0.05) of glutamate metabolism and pathways that require nitrogen from glutamine (Figure 3C).

Based on these results, we evaluated the effects of CB839, a small molecule glutaminase inhibitor currently being used in clinical trials, on Low-OXPHOS A375 and High-OXPHOS A375-R1 cells in vitro. CB839 significantly inhibited OCR in A375-R1 cells, but not in A375 cells (Figure 3D and E). CB839 treatment did not inhibit ECAR in the A375-R1, indicating that glycolysis did not depend on glutamine in the cell line (Supplementary Figure S3). A375-R1 cells were also more sensitive to growth inhibition (IC50 182 nM) by CB839 than A375 cells (IC50 437 nM) (Figure 3F). CB389 treatment significantly improved the OS of mice with A375-R1 intracranial xenografts (median OS 13 vs 5 days; *P* = .0292; Figure 3G). CB839 also improved OS of mice with intracranial xenografts of MEL624, a *BRAF-*mutant human melanoma cell line with High-OXPHOS and de novo resistance to MAPKi (median OS 31 vs 16 days; *P* = .0266; Figure 3H).^9,15^ No significant weight loss or toxicity was observed with CB839 treatment (Supplementary Figure S4), consistent with the favorable safety profile observed in patients.^28^

### Immune Features Associated With OXPHOS in MBMs

**Figure 4.**
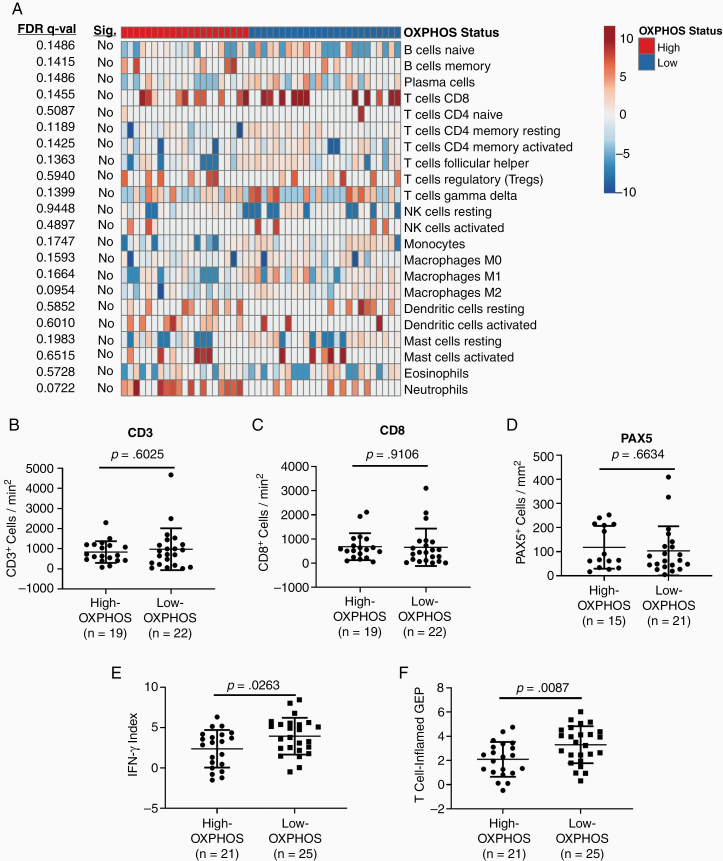
Oxidative phosphorylation associates with immunosuppression in melanoma brain metastases. (A) CIBERSORT analysis of High-OXPHOS (*n* = 21) and Low-OXPHOS (*n* = 25) MBMs. Data are presented as a heatmap of median-centered log2-tranformed estimates of the 22 immune cell populations listed on the right side of the heatmap. FDR q-values are listed to the left of the graph along with determination of significance (FDR q-val < 0.05). No immune cell population significantly differed between the groups. (B–D) IHC analysis for CD3-, CD8-, and PAX5-positive cells in High- and Low-OXPHOS MBMs. H-scores shown, as described in Figure 1. (E,F) Comparison of a 6-gene IFN-γ mRNA signature and 18-gene T-cell inflamed gene expression profile (GEP) for High- versus Low-OXPHOS MBMs. (B–F) Lines represent mean ± SD, and each dot represents a single tumor. Significance determined by 2-sided Student’s *t*-test.

In addition to increased OXPHOS, our previous analysis identified decreased immune cell infiltrates in MBMs compared to ECMs.^8^ Thus, we assessed if there are significant immunologic differences between High- and Low-OXPHOS MBMs. High-OXPHOS MBMs had significantly lower ImmuneScores, which is a metric calculated from the expression of 141 immune-related genes from numerous different immune cell subpopulations, compared to Low-OXPHOS MBMs (*P* = .0034;Supplementary Figure S5). Interestingly, CIBERSORT analysis of RNA-seq data identified no significant differences in any of 22 immune cell subpopulations (Figure 4A). Consistent with the CIBERSORT analysis, IHC staining showed no significant differences in CD3^+^ or CD8^+^ cells (T-cell markers) (Figure 4B and C) or PAX5^+^ cells (B-cell marker) (Figure 4D) between High- and Low-OXPHOS MBMs. While we did not observe significant differences in any of the immune cell subpopulation infiltrating the tumors, High-OXPHOS MBMs were characterized by significantly lower expression (*P* = .0263) of a 6-gene IFNγ mRNA signature that correlates with improved responsiveness to anti-PD1 immunotherapy^29^ (Figure 4E). Further, High-OXPHOS MBMs express significantly lower T cell–inflamed gene expression profile (GEP) (*P* = .0087) than Low-OXPHOS MBMs (Figure 4F). This larger signature, which also predicts response to anti-PD1, features IFNγ-responsive genes related to antigen presentation, chemokine expression, cytotoxic activity, and adaptive immune resistance.^29^

**Figure 5.**
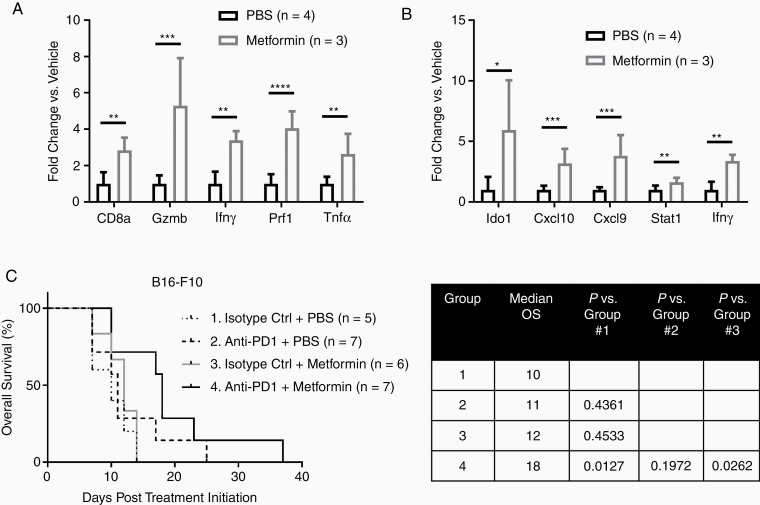
Metformin improves the response of High-OXPHOS, syngeneic intracranial melanoma xenografts to anti-PD1 immunotherapy. B16-F10 cells were implanted intracranially in C57BL/6 mice and treated for 96 h with low-dose metformin (50 mg/kg i.p. every other day; *n* = 3) or PBS (*n* = 4). qRT-PCR analysis was used to assess mRNA levels of (A) panel of genes previously shown to correlate with the presence of activated CD8^+^ T cells in melanomas, and (B) panel of IFNγ-related genes previously shown to predict response to anti-PD1 in metastatic melanoma patients. Values represent mean ± SD. ****P < .0001; ***P < .001; **P < .01, *P < .05 by 2-sided Student’s *t*-test. (C) Kaplan–Meier analysis of overall survival for C57BL/6 mice bearing intracranial B16-F10 xenografts treated with isotype antibody control (200 µg i.p. 3×/week) + PBS (10 µL/g body weight i.p. every other day) (Group 1; *n* = 5); anti-PD1 (200 µg i.p. 3×/week) + PBS (10 µL/g body weight i.p. every other day) (Group 2; *n* = 7); isotype control (200 µg i.p. 3×/week) + metformin (50 mg/kg i.p. every other day) (Group 3; *n* = 6); and anti-PD1 (200 µg i.p. 3×/week) + metformin (50 mg/kg i.p. every other day) (Group 4; *n* = 7). Significance of differences determined by log-rank testing.

In previous experiments using subcutaneous B16-F10 murine melanoma tumors, treatment with a low-dose metformin regimen inhibited OXPHOS exclusively in tumor cells and improved responsiveness to anti-PD1.^13^ To determine if metformin can also improve the immune microenvironment of MBMs, mice with intracranial luciferase-tagged B16-F10 tumors were treated with the low-dose metformin regimen (50 mg/kg i.p. every other day) or PBS for 96 h, and then were euthanized. qRT-PCR was performed on RNA isolated from the MBMs for a 5-gene signature indicative of activated CD8^+^ cytotoxic T cells, and 5 genes from the aforementioned 6-gene IFNγ mRNA signature (HLA-DRA is only found in humans and was therefore excluded).^29,30^ Metformin treatment significantly increased the expression of all 11 genes (*P* < .05 – *P* < .0001), consistent with improved immune functionality in the tumor microenvironment (Figure 5A and B). Additional mice were implanted intracranially with luciferase-tagged B16-F10 to test the therapeutic effects of metformin. After confirming tumor uptake, mice were treated with isotype control antibody, anti-PD1, metformin, or anti-PD1 with metformin. Only treatment with anti-PD1 and metformin improved OS versus control (median 18 vs 10 days; *P* = .0127; Figure 5C). Mice treated with the combination also had significantly improved OS compared to metformin alone (median 18 vs 12 days; *P* = .0262).While the combination quantitatively improved OS versus anti-PD1 alone, the result was not statistically significant (median OS 18 vs 11 days, *P* = .1972).

## Discussion

Previous studies by our group and others have implicated increased OXPHOS as a predictor of poor clinical outcomes^23,31^ and as a hallmark of brain metastases^8^ in patients with metastatic melanoma. Thus, there is a critical need to improve our understanding of the pathogenesis of this metabolic phenotype, and to develop rational, new therapeutic approaches to combat it. Our analyses of the unique cohort of surgically resected, molecularly characterized MBMs has demonstrated that OXPHOS is associated with significantly shorter OS, increased glutaminolysis, and impaired immune function. These results provide new insights into the pathogenesis of brain metastases and OXPHOS in this disease. Further, our functional studies support that targeting pathways and phenotypes associated with OXPHOS may be beneficial for MBMs.

The observed positive associations with PGC1α, MITF, and mTOR activity with OXPHOS levels in MBMs are consistent with our previous studies performed in melanoma cell lines.^9^ Notably, we previously showed that High-OXPHOS cell lines and xenografts were sensitive to combined treatment with the mTORC1/2i AZD2014 plus the MEKi AZD6244.^9^ However, here our pilot experiments showed that AZD2014 did not inhibit mTOR in intracranial xenografts, despite using a dose equivalent to twice the maximum tolerated dose in humans.^32^ Additional investigations are needed to evaluate additional dosing schedules with AZD2014 and/or other mTORC1/2 inhibitors as a therapeutic agents, but may be challenging to translate due to their toxicities. Of note, our recent work showed that treatment with the direct OXPHOS inhibitor IACS-010759 could inhibit mTOR signaling, suggesting a feedback circuit between these signaling and metabolic pathways.^15^

While we previously showed that IACS-010759 had single-agent activity in MBM models,^8^ results from phase I clinical testing and preclinical studies suggest that the toxicities observed with this agent may make clinical development challenging, particularly as a combinatorial strategy.^14,15^ Thus, there is a strong rationale to identify other targetable dependencies of High-OXPHOS MBMs. Interestingly, our analysis of clinical and preclinical samples demonstrates utilization of, and dependence upon, glutamine metabolism in High-OXPHOS MBMs. Our RNA-based studies demonstrated increased activity of multiple glutamine-dependent metabolic pathways in clinical samples, and direct metabolite analysis of High- and Low-OXPHOS preclinical MBM models confirmed increased glutamine metabolism in High-OXPHOS MBMs. These results are consistent with work by Baenke et al., which showed that melanomas with acquired resistance to BRAF inhibitors with High-OXPHOS switched from glucose to glutamine as their primary energy source.^27^ Excitingly, CB839, a novel small molecule glutaminase inhibitor currently being used in multiple clinical trials, improved survival as a single-agent in 2 different models of High-OXPHOS, MAPKi-resistant MBMs (A375-R1 and MEL624). The favorable toxicity profile of CB839 supports the feasibility of evaluating combinatorial strategies, in addition to single-agent activity. Notably, clinical trials conducted to date in melanoma with CB839 have excluded patients with active CNS disease,^28^ though our results suggest this agent could particularly benefit patients with MBMs. Indeed, our results here and previously strongly support that both preclinical and clinical investigations of strategies targeting OXPHOS and associated pathways should include evaluation of brain metastases. Additional studies are also needed to further characterize the effects of glutaminase inhibition in melanoma, including further characterization of metabolic effects and the impact on MBMs with relatively Low OXPHOS.

Finally, our analyses demonstrate that OXPHOS is associated with features consistent with functional immunosuppression in MBMs, including significantly decreased expression of gene signatures that predict response to anti-PD1 immunotherapy.^29^ These findings are consistent with the decreased responsiveness observed with pembrolizumab and with nivolumab in MBM patients.^4,5^ The OXPHOS-associated decrease in immune activation without changes in the degree of immune cell subpopulation infiltration is also consistent with recent analyses of biopsies of ECMs with increased OXPHOS from metastatic melanoma patients receiving anti-PD1 treatment.^12^ Notably, in preclinical models, it was previously shown that OXPHOS inhibition can enhance response to anti-PD1 immunotherapy in subcutaneous xenografts,^12,13^ which we also observed in our experiments with B16-F10 MBMs. Together our results are consistent with growing data implicating aberrant metabolism in resistance to immunotherapy in melanoma, and they are the first to directly implicate OXPHOS as a mediator of immunosuppression in MBMs. Overall the results strongly support the rationale to further explore strategies combining metabolic inhibitors with anti-PD1, including in additional murine and/or humanized MBM mouse models. Furthermore, the results highlight the critical need to include, not exclude, patients with brain metastases in such clinical trials.

## Supplementary Material

vdaa177_suppl_Supplementary_Material

## Notes

**Conflict of interest statement**. M.A.D. has been a consultant to Roche/Genentech, Array, Novartis, BMS, GSK, Sanofi-Aventis, Vaccinex and Apexigen, and he has been the PI of research grants to MD Anderson by Roche/Genentech, GSK, Sanofi-Aventis, Merck, Myriad, and Oncothyreon. J.L.M. has been a consultant for Roche/Genentech, BMS, and Merck. M.T.T. has been a consultant Novartis, Myriad Genetics, and Seattle Genetics. A.J.L. has been a consultant for BMS, GSK/Novartis, Roche/Genentech/Ignyta, MedImmune/AstraZeneca, Bayer/Loxo, Merck, Guardant, and ArcherDX.

## Notes

**Authorship Statement.** Concept and design: G.M.F. and M.A.D. Acquisition of data: G.M.F., R.A.G., Q.H., A.Y.J., L.E.H., B.K., C.W.H., A.J.L., M.T.T., and MAD. Analysis and interpretation of data: G.M.F., A.Y.J., and M.A.D. Writing, review, and/or revision of the manuscript: All authors. Study supervision: G.M.F. and M.A.D.
