# Supplementary material for: Clinical, molecular, metabolic, and immune features associated with oxidative phosphorylation in melanoma brain metastases
Source: Neurooncol Adv. 2021 Jan 6;3(1):vdaa177. doi: 10.1093/noajnl/vdaa177 (PMC7865080; doi:10.1093/noajnl/vdaa177)
Supplement: vdaa177_suppl_Supplementary_Material [file vdaa177_suppl_supplementary_material.pdf]

Supplemental Tables and Figures

Tables S1-4

Figures S1-5

Table S1. Characteristics of Cell Lines. OCR, oxygen consumption rate. ECAR, extracellular acidification rate.

Cell Line	Species	Driver Mutations	OXPHOS Status	Basal OCR (Ratio to A375)	Basal ECAR (Ratio to A375)
**A375**	Human	$BRAF^{V600E}$	Low	1.0	1.0
**A375-R1**	Human	$BRAF^{V600E}$;$MEK1^{F129L}$	High	3.1	1.0
**MEL624**	Human	$BRAF^{V600E}$	High	1.8	0.9
**B16-F10**	Mouse	---	High	2.7	1.6

Table S2. Gene Sets Comprising the OXPHOS-Index Signature

Gene Set	MSigDB Database
HALLMARK_OXIDATIVE_PHOSPHORYLATION	Hallmarks
KEGG_OXIDATIVE_PHOSPHORYLATION	C2: Curated Gene Sets
KEGG_CITRATE_CYCLE_TCA_CYCLE	C2: Curated Gene Sets
REACTOME_TCA_CYCLE_AND_RESPIRATORY_ELECTRON_TRANSPORT	C2: Curated Gene Sets
REACTOME_CITRIC_ACID_CYCLE_TCA_CYCLE	C2: Curated Gene Sets
REACTOME_RESPIRATORY_ELECTRON_TRANSPORT_ATP_SYNTHESIS_BY_CHEMIOSMOTIC_COUPLING_AND_HEAT_PRODUCTION_BY_UNCOUPLING_PROTEINS	C2: Curated Gene Sets
MOOTHA_TCA	C2: Curated Gene Sets
MOOTHA_VOXPHOS	C2: Curated Gene Sets
OPIndex → arithmetic mean of ssGSEA scores for listed gene sets	

Table S3. Comparison in Mutation Proportion Between High- (N=8) and Low- (N=5) OXPHOS MBMs

Gene	P Value
**ABL1**	1
**AKT1**	1
**ALK**	1
**AR**	1
**ATM**	1
**ATR**	1
**AURKA**	1
**BAP1**	1
**BRAF.any**	1
**BRAF.nonV600**	1
**BRAF.V600**	1
**BRCA1**	0.38
**BRCA2**	1
**CDK4**	1
**CDK6**	1
**CDKN2A**	1
**CHEK2**	1
**CSF1R**	1
**DDR1**	1
**DDR2**	1
**DNMT3A**	1
**EGFR**	1
**ESR1**	1
**FGFR1**	1
**FGFR2**	1
**FGFR3**	0.38
**FLT1**	1
**FLT4**	1
**GNAQ**	1
**HDAC9**	1
**IDH1**	1
**IDH2**	1
**IGF1R**	1
**JAK1**	1
**JAK3**	1
**KDR**	1
**KIT**	1
**MAP2K1**	1
**MAP2K4**	1
**MAP3K1**	0.49
**MAP3K4**	0.49
**MET**	0.38
**MITF**	0.38
**MPL**	1
**MTOR**	1
**NF1**	1
**NF2**	1
**NOTCH1**	1
**NOTCH2**	1
**NOTCH3**	1
**NOTCH4**	1
**NRAS**	0.49
**PALB2**	1
**PDGFRA**	0.49
**PDGFRB**	1
**PIK3CA**	1
**PIK3R1**	0.38
**PTCH1**	1
**PTEN**	1
**PTPN11**	1
**RB1**	1
**RET**	1
**SMO**	1
**STK11**	1
**SYK**	0.13
**TET2**	1
**TP53**	0.38
**TSC1**	1
**TSC2**	1

Table S2. The prevalence of non-synonymous somatic mutations in 74 clinically relevant genes was compared between High- (N=8) and Low- (N=5) OXPHOS MBMs with available WES data. Gene names are listed in bold. The *BRAF* gene results (any mutation, nonV600 mutation, and V600 mutation) is highlighted in red. No genes met the criteria for statistical significance ($p<0.05$) via Fisher's exact test.

Table S4. KEGG Metabolism Gene Sets Used in Metabolomics Pathway Analyses

Pathway Name	Pathway Name
GLYCOLYSIS GLUCONEOGENESIS	GLYCOSAMINOGLYCAN BIOSYNTHESIS CHONDROITIN SULFATE
CITRATE CYCLE TCA CYCLE	GLYCOSAMINOGLYCAN BIOSYNTHESIS KERATAN SULFATE
PENTOSE PHOSPHATE PATHWAY	GLYCOSAMINOGLYCAN BIOSYNTHESIS HEPARAN SULFATE
PENTOSE AND GLUCURONATE INTERCONVERSIONS	GLYCEROLIPID METABOLISM
FRUCTOSE AND MANNOSE METABOLISM	INOSITOL PHOSPHATE METABOLISM
GALACTOSE METABOLISM	GLYCOSYLPHOSPHATIDYLINOSITOL GPI ANCHOR BIOSYNTHESIS
ASCORBATE AND ALDARATE METABOLISM	GLYCEROPHOSPHOLIPID METABOLISM
FATTY ACID METABOLISM	ETHER LIPID METABOLISM
STEROID BIOSYNTHESIS	ARACHIDONIC ACID METABOLISM
PRIMARY BILE ACID BIOSYNTHESIS	LINOLEIC ACID METABOLISM
STEROID HORMONE BIOSYNTHESIS	ALPHA LINOLENIC ACID METABOLISM
OXIDATIVE PHOSPHORYLATION	SPHINGOLIPID METABOLISM
PURINE METABOLISM	GLYCOSPHINGOLIPID BIOSYNTHESIS LACTO/NEOLACTO SERIES
PYRIMIDINE METABOLISM	GLYCOSPHINGOLIPID BIOSYNTHESIS GLOBO SERIES
ALANINE ASPARTATE AND GLUTAMATE METABOLISM	GLYCOSPHINGOLIPID BIOSYNTHESIS GANGLIO SERIES
GLYCINE SERINE AND THREONINE METABOLISM	PYRUVATE METABOLISM
CYSTEINE AND METHIONINE METABOLISM	GLYOXYLATE AND DICARBOXYLATE METABOLISM
VALINE LEUCINE AND ISOLEUCINE DEGRADATION	PROPANOATE METABOLISM
VALINE LEUCINE AND ISOLEUCINE BIOSYNTHESIS	BUTANOATE METABOLISM
LYSINE DEGRADATION	ONE CARBON POOL BY FOLATE
ARGININE AND PROLINE METABOLISM	RIBOFLAVIN METABOLISM
HISTIDINE METABOLISM	NICOTINATE AND NICOTINAMIDE METABOLISM
TYROSINE METABOLISM	PANTOTHENATE AND COA BIOSYNTHESIS
PHENYLALANINE METABOLISM	FOLATE BIOSYNTHESIS
TRYPTOPHAN METABOLISM	RETINOL METABOLISM
BETA ALANINE METABOLISM	PORPHYRIN AND CHLOROPHYLL METABOLISM
TAURINE AND HYPOTAURINE METABOLISM	TERPENOID BACKBONE BIOSYNTHESIS
SELENOAMINO ACID METABOLISM	LIMONENE AND PINENE DEGRADATION
GLUTATHIONE METABOLISM	NITROGEN METABOLISM
STARCH AND SUCROSE METABOLISM	SULFUR METABOLISM
N GLYCAN BIOSYNTHESIS	AMINOACYL TRNA BIOSYNTHESIS
OTHER GLYCAN DEGRADATION	METABOLISM OF XENOBIOTICS BY CYTOCHROME P450
O GLYCAN BIOSYNTHESIS	DRUG METABOLISM CYTOCHROME P450
AMINO SUGAR AND NUCLEOTIDE SUGAR METABOLISM	DRUG METABOLISM OTHER ENZYMES
GLYCOSAMINOGLYCAN DEGRADATION	BIOSYNTHESIS OF UNSATURATED FATTY ACIDS

Figure S1

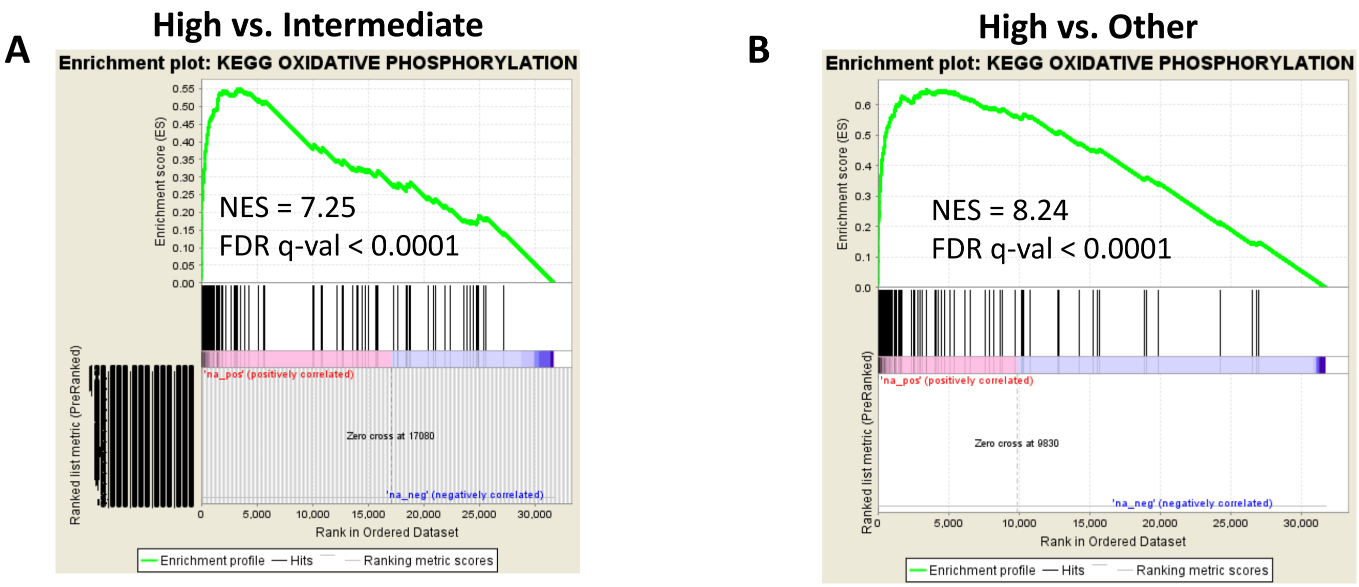

Figure S1. Pathway analysis of MBMs clustered by OXPHOS gene set expression. (**A**) Preranked Gene Set Enrichment Analysis (GSEA-P) demonstrating enrichment of OXPHOS in MBMs identified as "High-OXPHOS MBMs" vs. "Intermediate-OXPHOS MBMs." (**B**) GSEA-P demonstrating enrichment of OXPHOS in "High-OXPHOS MBMs" vs. all other MBMs. (**A-B**) Normalized enrichment score (NES) and FDR q-val are listed on the enrichment plots.

Figure S2

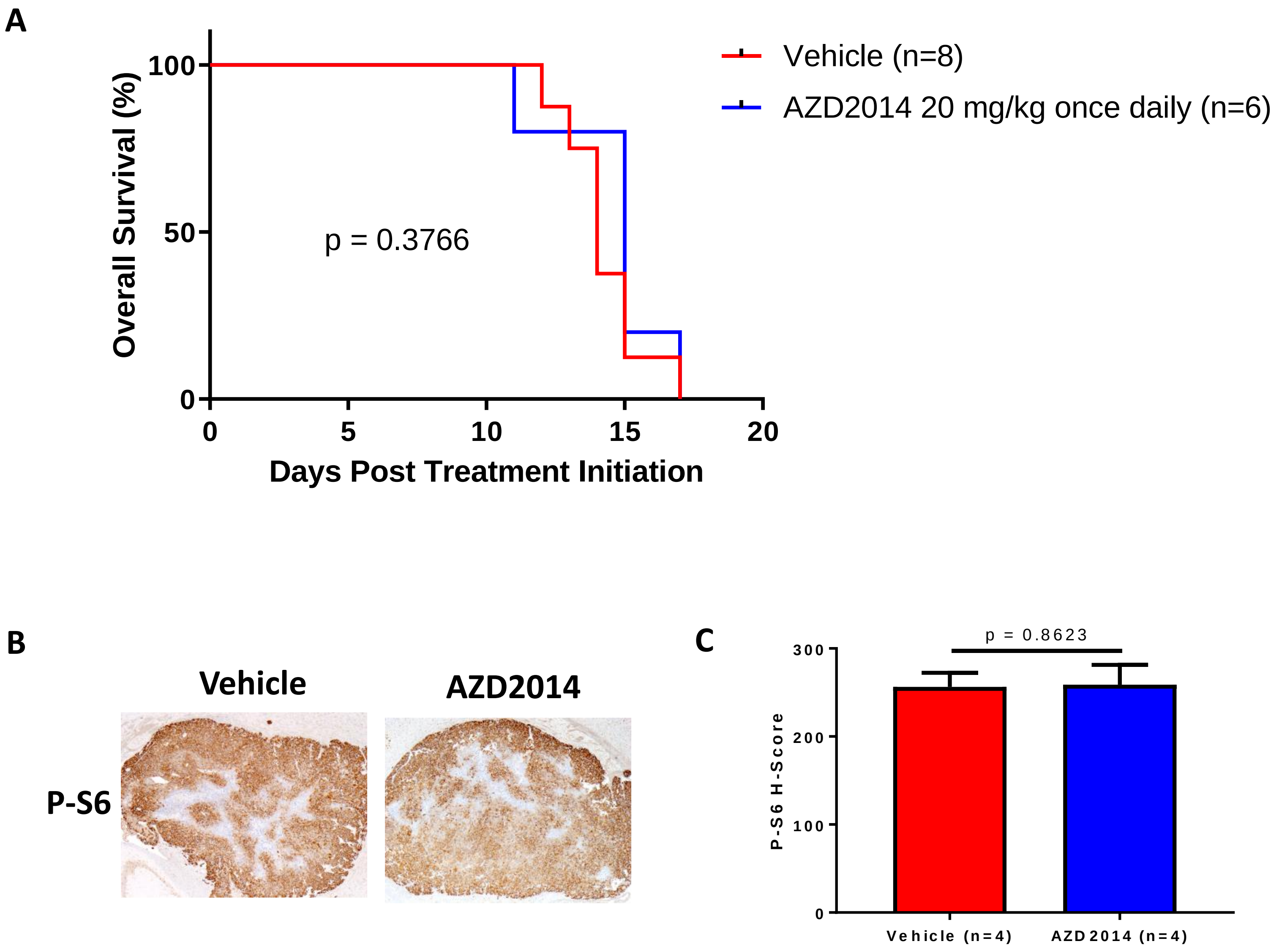

Figure S2: Pharmacodynamics of AZD2014 in High-OXPHOS, MAPKi-resistant intracranial melanoma xenografts. (**A**) Kaplan-Meier analysis of overall survival (OS) of mice bearing intracranial (ICr) A375-R1 xenografts treated with either AZD2014 (20 mg/kg p.o. once daily; Blue line) or vehicle (Red line). Significance was determined by log-rank testing. (**B**) Representative P-S6 staining of ICr A375-R1 xenografts treated for 1 week with AZD2014 (20 mg/kg p.o. once daily) or vehicle. (**C**) Average H-scores for P-S6 staining analysis of ICr A375-R1 xenografts treated for 1 week with either AZD2014 (20 mg/kg p.o. once daily) or vehicle. Average values and S.D. of four biological replicates per condition are displayed. Significance determined by two-sided Student's *t*-test.

Figure S3

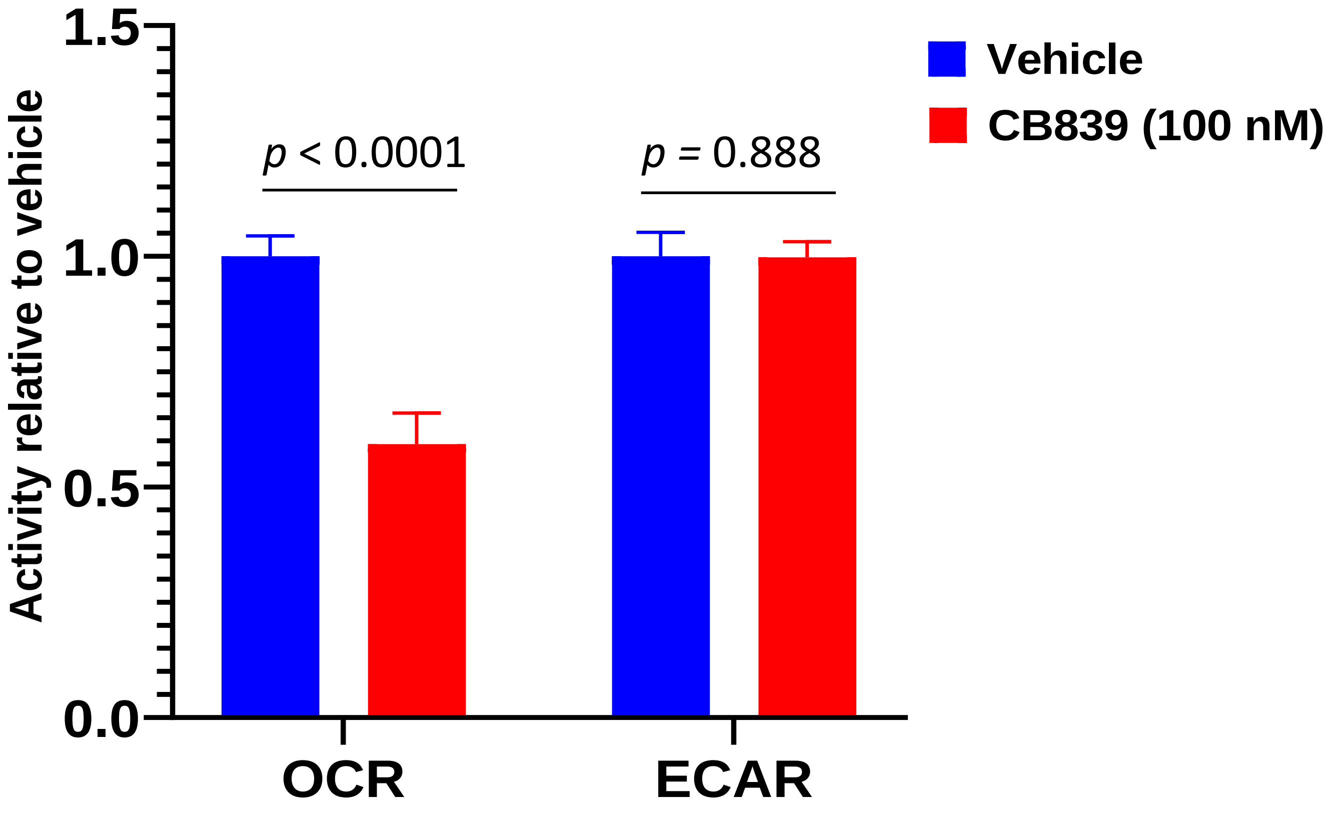

Figure S3. Effects of CB839 on oxidative phosphorylation and glycolysis in High-OXPHOS A375-R1 cells *in vitro.* A375-R1 human melanoma cell lines were treated 12 hours with vehicle (*Blue*) or CB-839 100 nM (*Red*) *in vitro.* Seahorse Bioanalyzer analysis was performed to assess basal oxygen consumption rate (OCR) and extracellular acidification rate (ECAR). OCR and ECAR were normalized to average value for vehicle-treated cells. Bars show average of biological replicates (n=12), error bars show standard deviation, significance determined by Student's t-test. Treatment with CB839 significantly reduced OCR (oxidative phosphorylation) but not glycolysis (ECAR) in the High-OXPHOS A375-R1.

Figure S4

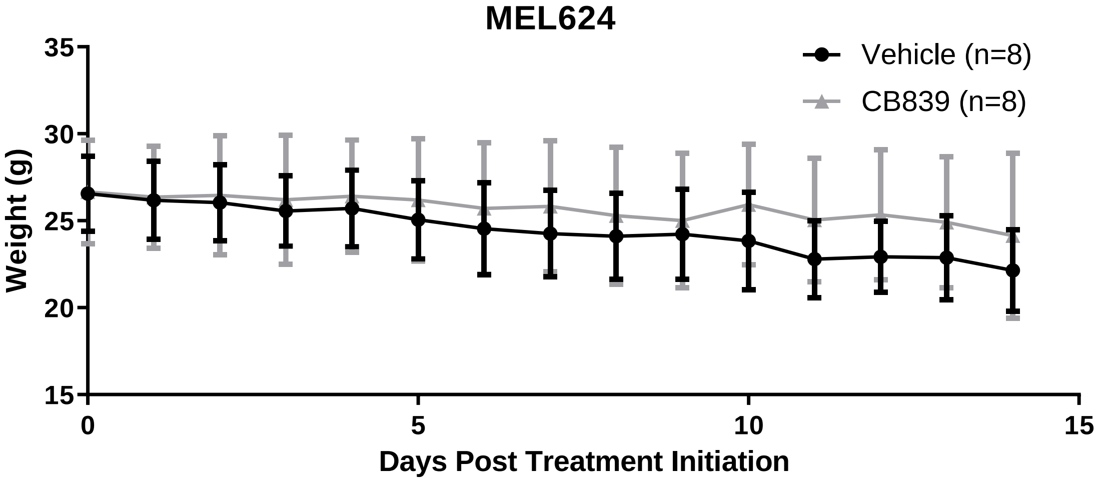

Figure S4. Tolerability of the glutaminase inhibitor CB839 in mice bearing intracranial High-OXPHOS, MAPKi-resistant melanoma xenografts. Assessment of body weight for mice bearing intracranial MEL624 xenografts and treated with vehicle or CB839 (200 mg/kg p.o. twice daily). Mice were weighed every 2 days throughout the experiment. Weights for the first 14 days of treatment are displayed. Values represent mean ± S.D.

Figure S5

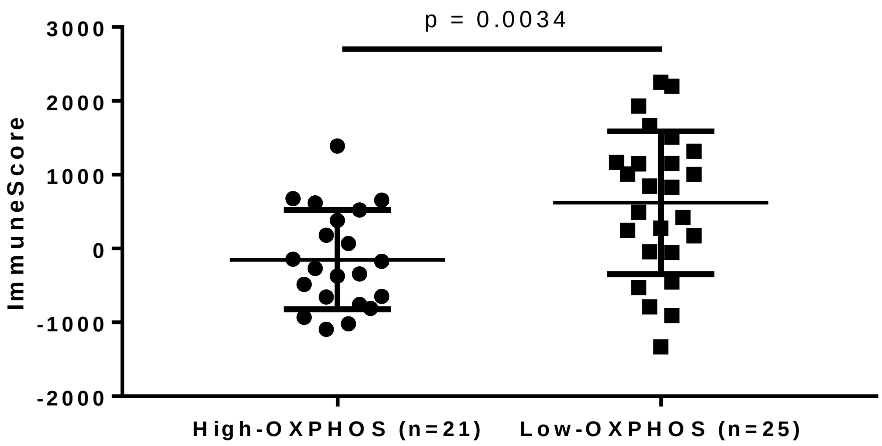

Figure S5. Association of OXPHOS with ESTIMATE ImmuneScore output. ESTIMATE ImmuneScore analysis of High-OXPHOS (n=21) and Low-OXPHOS (n=25) MBMs. Lines represent mean ± S.D., and each dot represents a single tumor. Significance determined by two-sided Student's *t*-test.
